# Genomic analysis of circulating tumor cells in adenosquamous carcinoma of the prostate: a case report

**DOI:** 10.1186/s12920-021-01068-w

**Published:** 2021-09-03

**Authors:** Junji Kitamura, Satoru Taguchi, Takatsugu Okegawa, Kazuki Honda, Toshihiko Kii, Yoshihiro Tomida, Ryuki Matsumoto, Naoki Ninomiya, Kazuki Masuda, Yu Nakamura, Tsuyoshi Yamaguchi, Manami Kinjo, Mitsuhiro Tambo, Aya Isomura, Akimasa Hayashi, Hiroshi Kamma, Eiji Higashihara, Junji Shibahara, Hiroshi Fukuhara

**Affiliations:** 1 Department of Urology, Kyorin University School of Medicine, 6-20-2 Shinkawa, Mitaka, Tokyo, 181-8611 Japan; 2 Department of Pathology, Kyorin University School of Medicine, Tokyo, Japan; 3 Department of ADPKD Research, Kyorin University School of Medicine, Tokyo, Japan

**Keywords:** Adenosquamous carcinoma, Case report, Circulating tumor cell, Liquid biopsy, Prostate cancer

## Abstract

**Background:**

Adenosquamous carcinoma of the prostate (ASCP) is an extremely rare and aggressive prostate cancer variant, whose genomic characteristics have not been elucidated. Although liquid biopsy of circulating tumor cells (CTCs) is an emerging topic in oncology, no study has assessed CTCs in patients with ASCP.

Case presentation.

A 76-year-old man presented with discomfort in his urethra. His prostate-specific antigen (PSA) level was 13.37 ng/mL. A computed tomography (CT) scan indicated a prostate mass with multiple lymph node and lung metastases. The patient underwent transurethral resection of the prostate and prostatic needle biopsy; both specimens demonstrated Gleason grade group 5 acinar adenocarcinoma of the prostate. Bone scintigraphy indicated bone metastasis in the ischium. Combined androgen blockade was implemented, and his serum PSA level rapidly decreased to 0.01 ng/mL. However, a CT scan 6 months after the initial diagnosis revealed worsening of the disease. The patient therefore underwent repeated prostatic needle biopsy; its specimen demonstrated prostatic adenocarcinoma together with squamous carcinoma components. As immunohistochemical analyses showed the tumor cells to be negative for CD56, chromogranin A, synaptophysin, and PSA, the definitive diagnosis was ASCP. Although the patient underwent chemotherapy (docetaxel and cabazitaxel), he died of the disease 3 months after the diagnosis of ASCP, or 13 months after the initial diagnosis of prostatic adenocarcinoma. His PSA values remained ≤ 0.2 ng/mL. CTCs from the patient’s blood (collected before starting docetaxel) were analyzed and genomically assessed. It showed 5 cytokeratin (CK)^+^ CTCs, 14 CK^−^ CTCs, and 8 CTC clusters, per 10 mL. Next-generation sequencing identified a total of 14 mutations in 8 oncogenes or tumor suppressor genes: *PIK3CB*, *APC*, *CDKN2A*, *PTEN*, *BRCA2*, *RB1*, *TP53*, and *CDK12*. Of 14 mutations, 9 (64%) were detected on CK^−^ CTCs and 5 (36%) were detected on CK^+^ CTCs.

**Conclusions:**

This is the first report of CTC analysis and genomic assessment in ASCP. Although the prognosis of ASCP is dismal due to lack of effective treatment, genomic analysis of CTCs might lead to effective treatment options and improved survival.

## Background

Adenosquamous carcinoma of the prostate (ASCP) is an extremely rare and aggressive prostate cancer (PC) variant [1], with an age-adjusted incidence rate of 0.03 cases per million per year [2]. A comprehensive survey using the Surveillance, Epidemiology and End Results (SEER) database between 1973 and 2008 detected only 27 cases of ASCP [2]. ASCP was first described by Thompson in 1942 [3], and is defined by the presence of both glandular and squamous components [4, 5]. Among published ASCP cases [4–25], two-thirds of patients had had prior androgen deprivation therapy (ADT) and/or radiation therapy for acinar adenocarcinoma of the prostate [4–6, 8–10, 13, 16, 17, 22], whereas the remaining one-third of patients were considered de novo cases [7, 11, 12, 14, 15, 18–21, 23–25]. Owing to its extreme rarity, little is known about genomic characteristics of ASCP [13, 14, 26].

Liquid biopsy of, for example, circulating tumor cells (CTCs) and cell-free DNA is an emerging topic in oncology, and has been actively investigated in patients with PC [27–29]. However, no study has assessed CTCs in patients with ASCP, probably because of its extreme rarity. The present study aimed to conduct a genomic analysis of CTCs in a patient with ASCP, developed after primary ADT for Gleason grade group 5 PC.

## Case presentation

**Fig. 1:**
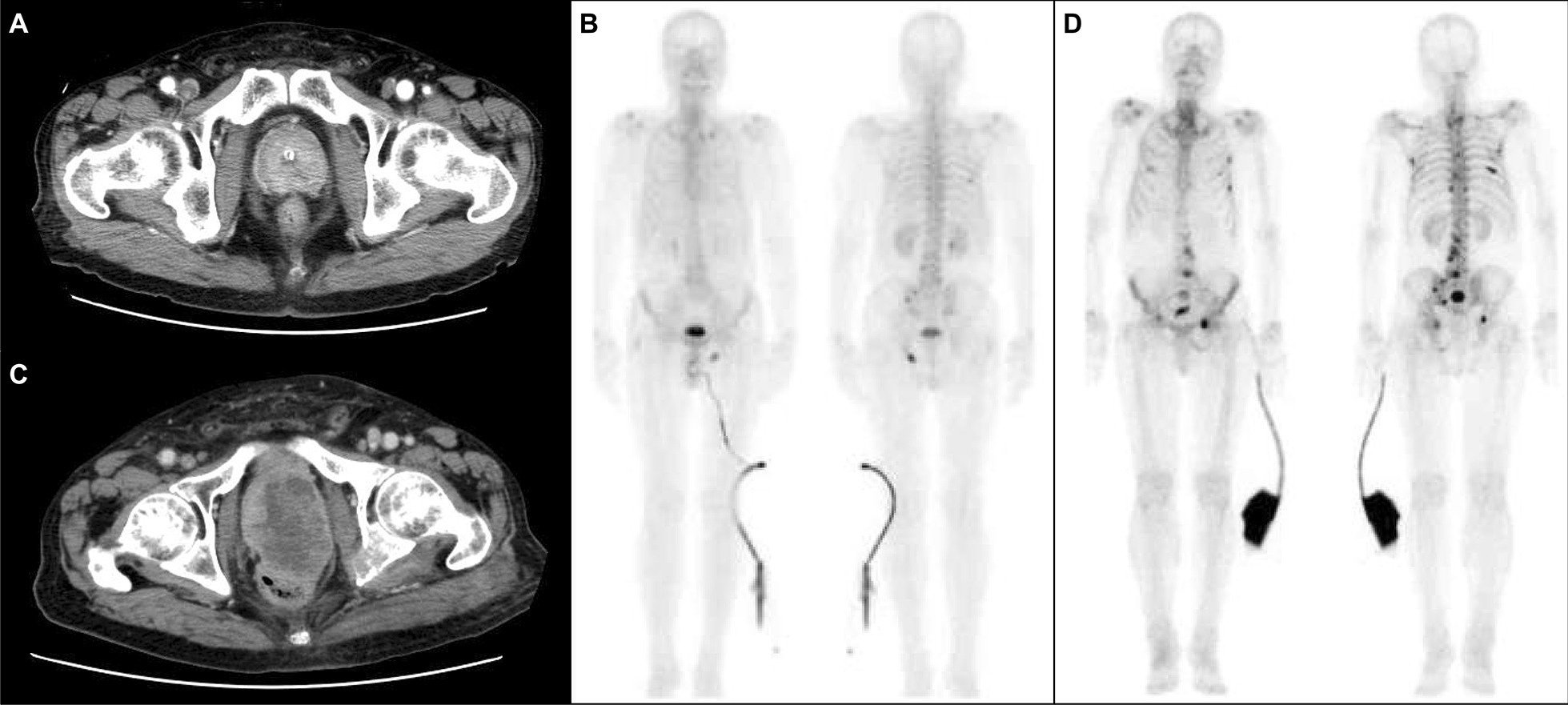
Images of **a** CT scan and **b** bone scintigraphy at the initial diagnosis of Gleason grade group 5 PC (PSA: 13.37 ng/mL), and those of **c** CT scan and **d** bone scintigraphy at the diagnosis of ASCP (PSA: 0.02 ng/mL), respectively. During the course, the prostatic mass grew remarkably (a → c) and the number of bone metastases increased (b → d), despite maintaining a low PSA level. Abbreviations: ASCP, adenosquamous carcinoma of the prostate; CT, computed tomography; PC, prostate cancer; PSA, prostate-specific antigen

**Fig. 2:**
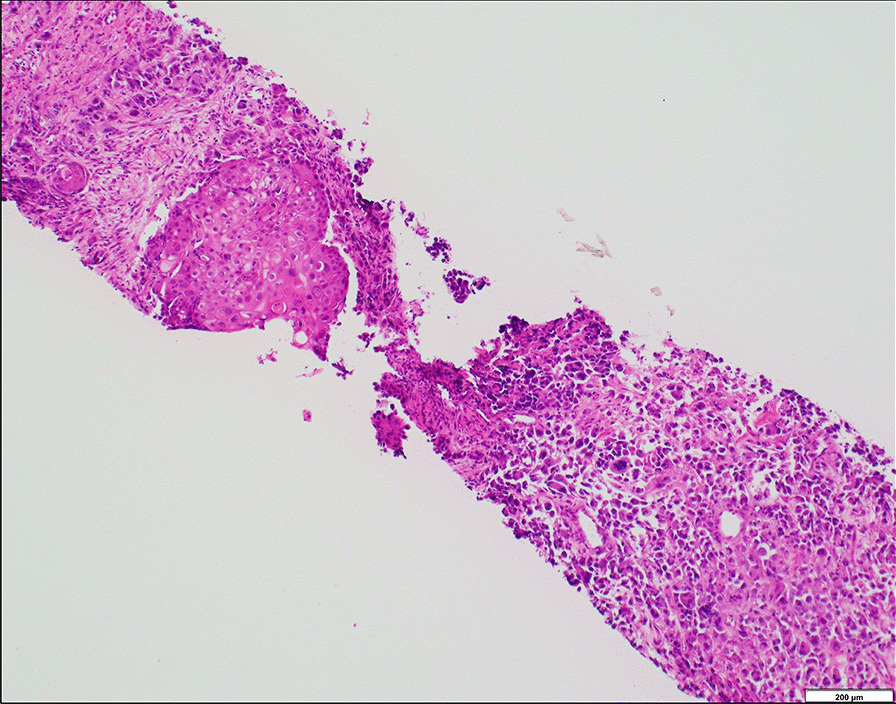
A microscopic image of repeated prostatic needle biopsy (hematoxylin and eosin stain), showing adenocarcinoma components after treatment with ADT (therapeutic effect was judged to be Grade 1), together with squamous carcinoma components. The image was obtained using the following equipment and software: microscope, BX53; objective lens, UPLXAPO; camera, DP27; adapter, U-TV1XC; and acquisition software, cellSens (Olympus Corporation, Japan). The measured size and resolution at which the image was acquired were 2448 × 1920 pixels and 96 pixels per inch, respectively. Abbreviation: ADT, androgen deprivation therapy

A 76-year-old man presented to a local hospital with discomfort in his urethra. His prostate-specific antigen (PSA) level was 13.37 ng/mL. He had undergone percutaneous coronary intervention for angina pectoris 3 years before. Based on a computed tomography (CT) scan that indicated a prostatic mass (Fig. 1a), along with bilateral hydronephrosis, multiple lymph node metastases, and multiple lung metastases, the patient immediately underwent transurethral resection of the prostate and prostatic needle biopsy; both specimens showed diffusely proliferating acinar adenocarcinoma with Gleason scores of 5 + 4 = 9 and 5 + 5 = 10 (grade group 5). Technetium-99m bone scintigraphy indicated bone metastasis in the ischium (Fig. 1b). Tumor markers other than PSA, including serum squamous cell carcinoma antigen and neuron-specific enolase, were within normal limits. Primary ADT with luteinizing hormone-releasing hormone analogue plus bicalutamide (combined androgen blockade) was implemented. Although his serum PSA level rapidly decreased to 0.01 ng/mL, radiological assessments (CT scan and bone scintigraphy) conducted 6 months after the initial diagnosis revealed worsening of the primary site (Fig. 1c), and bone metastases in the ribs, vertebrae, pelvic bones, and the skull (Fig. 1d). After undergoing percutaneous cystostomy due to urinary retention, the patient was referred to our hospital for further evaluation and treatment. Significant laboratory investigations showed an elevated serum squamous cell carcinoma antigen (30.2 ng/mL; normal range: 0–1.5 ng/mL) and neuron-specific enolase (41.2 ng/mL; normal range: 0–16.3 ng/mL). Serum PSA and testosterone levels were maintained at 0.02 ng/mL and < 0.03 ng/mL, respectively. Repeated prostatic needle biopsy was performed, the specimen of which showed prostatic adenocarcinoma after treatment with ADT (the therapeutic effect was judged to be Grade 1) together with squamous carcinoma components (Fig. 2). Immunohistochemical analyses revealed that the tumor cells were negative for CD56, chromogranin A, synaptophysin, and PSA. Accordingly, the definitive diagnosis was ASCP. After undergoing a transverse colostomy for rectal invasion of the prostatic tumor, and palliative irradiation for neck and pelvic bone metastases (8 Gy/1 Fr), the patient began docetaxel chemotherapy (70 mg/m^2^). However, a CT scan just a month after initiating chemotherapy showed local progression of the prostatic tumor with invasion to the cavernous body of the penis and worsening of multiple lung and bone metastases. He then switched to cabazitaxel chemotherapy (20 mg/m^2^), along with pegfilgrastim for primary prophylaxis for febrile neutropenia. However, he needed to immediately discontinue it because of septic shock caused by scrotal abscess due to tumor invasion. Although he recovered from septic shock following scrotal debridement and antibiotic treatment, he eventually died 3 months after the diagnosis of ASCP, or 13 months after the initial diagnosis of prostatic adenocarcinoma. We followed the patient’s PSA values every month, but they remained at ≤ 0.2 ng/mL.

CTC analysis along with genomic assessment was performed using the patient’s blood sample, collected before starting docetaxel chemotherapy. The protocol of the CTC analysis was approved by the Institutional Review Board of Life Science Research Laboratory, Tosoh Corporation (Protocols LS18-02 and LS19-02) and Kyorin University School of Medicine (approval number 1205). The patient provided written informed consent prior to sample collection. Because we will report the details of this CTC analysis in another paper, which is currently under preparation, we will provide only a brief description of the procedure here. First, red blood cell lysis was performed on the patient’s blood sample, using a lysing solution. Second, negative enrichment of the tumor cells was conducted by depleting the white blood cells, using CD45 and CD15 Dynabeads (Thermo Fisher Scientific, US). Third, the suspension of tumor cell-enriched mononucleated cells was loaded into the CTC detection device, and a suitable voltage was applied to entrap cells in the microwells, using dielectrophoretic force [30]. Fourth, the cell chambers were immunostained for cytokeratin (CK, an epithelial marker) and CD45 (a white blood cell marker), and with diamidino-2-phenylindole (DAPI, a nuclear stain). Images of immunofluorescent staining of each microwell were captured and analyzed using a custom-made software. Fifth, tumor cells were isolated from the microwells by a micromanipulated aspirator and released into respective tubes. Last, for the genomic analysis of isolated single tumor cells, whole-genome amplification (WGA) was carried out with the Ampli1™ WGA Kit (Silicon Biosystems, Bologna, Italy). After confirming success of the WGA by gel electrophoresis, next-generation sequencing (NGS) was conducted using the Ion Ampliseq™ Cancer Hotspot Panel v2 (Thermo Fisher Scientific), which can survey hotspot regions of 50 oncogenes and tumor suppressor genes. The clinical significance of detected mutations (i.e. variant classification) was assessed using multiple public databases (e.g. ClinVar, OncoKB, etc.) in a comprehensive manner.

Table 1 summarizes results of CTC analysis and genomic analysis of CTCs in the present case. The specimen showed 5 CK^+^ CTCs, 14 CK^−^ CTCs, and 8 CTC clusters per 10 mL (Table 1A). The NGS identified a total of 14 mutations in 8 oncogenes or tumor suppressor genes: *PIK3CB*, *APC*, *CDKN2A*, *PTEN*, *BRCA2*, *RB1*, *TP53*, and *CDK12*. Of 14 mutations, 9 (64%) were detected on CK^−^ CTCs and 5 (36%) were detected on CK^+^ CTCs. Five of 14 (36%) mutations showed an allele frequency of > 20% (Table 1B).

**Table 1:** Results of (A) CTC analysis and (B) genomic analysis of CTCs in this case

(A)	
CTC type	Number of detections
CTC-CK^+^	5 CTCs/10 mL
CTC-CK^−^	14 CTCs/10 mL
CTC-cluster	8 clusters/10 mL

(B)								
Chromosome	Position	Reference	Alteration	Gene	Type of variant, protein change (if any)	Variant classification	CK status	Allele frequency (%)
3	138699065	A	T	PIK3CB	Missense variant, p.L50M, p.L538M	VUS	CK^+^	5.2
**3**	**138699162**	**T**	**G**	**PIK3CB**	**Intronic variant**	**VUS**	**CK**^**−**^	**21.1**
5	112840058	ATT	A	APC	Frameshift variant, p.L1471fs, p.L1489fs	LP	CK^+^	10.5
**9**	**21974504**	**G**	**T**	**CDKN2A**	**Intronic variant**	**VUS**	**CK**^**−**^	**47.3**
10	87925549	ATACAATC	A	PTEN	Frameshift variant, p.Y68fs, p.Y241fs	VUS	CK^−^	18.4
10	87933078	G	T	PTEN	Missense variant, p.D107Y, p.D280Y	LP	CK^−^	5.3
**13**	**32362653**	**T**	**A**	**BRCA2**	**Missense variant, p.C2646S**	**VUS**	**CK**^**+**^	**31.6**
**13**	**48381316**	**T**	**A**	**RB1**	**Stop-gain variant, p.L523X**	**LP**	**CK**^**−**^	**52.6**
17	7673794	C	T	TP53	Missense variant, p.A144T, p.A117T, p.A237T, p.A276T	LP	CK^+^	2.6
17	7673799	A	G	TP53	Missense variant, p.V142A, p.V115A, p.V235A, p.V274A	LP	CK^−^	15.7
17	7675161	G	C	TP53	Missense variant, p.P19A, p.P112A, p.P151A	LP	CK^−^	18.4
17	7676397	AG	A	TP53	Frameshift variant, p.P27fs	LP	CK^−^	13.2
**17**	**7687968**	**C**	**A**	**TP53**	**Intronic variant**	**VUS**	**CK**^**−**^	**28.9**
17	39531218	T	TG	CDK12	Frameshift variant, p.W1450fs, p.W1459fs	LP	CK^+^	7.9

**Bold**: Allele frequency > 20%

Abbreviations: CK, cytokeratin; CTC, circulating tumor cell; LP, likely pathogenic; VUS, variant of uncertain significance

## Discussion and conclusion

To our knowledge, this is the first report of analysis and genomic assessment of CTCs in a patient with ASCP. ASCP is an extremely rare, aggressive variant of PC [1], with an age-adjusted incidence rate of 0.03 cases per million per year [2]. A comprehensive survey using the SEER database between 1973 and 2008 detected only 27 cases of ASCP [2]. According to a recent analysis on rare histological variants of PC using the National Cancer Database, ASCP accounted for 0.003% (*n* = 45) of all cases (*n* = 1,345,618) with PC diagnosed between 2004 and 2015 [1]. ASCP is histologically defined by the presence of both glandular and squamous components [4, 5]. Two-thirds of ASCP patients reportedly had had prior ADT and/or radiation therapy for acinar adenocarcinoma of the prostate [4–6, 8–10, 13, 16, 17, 22], whereas the remaining one-third of patients were considered de novo cases [7, 11, 12, 14, 15, 18–21, 23–25]. The present case developed after primary ADT for Gleason grade group 5 PC.

Because of its extreme rarity, little is known about the molecular genetics of ASCP [13, 14, 26]. An early study reported that both glandular and squamous components showed strong immunohistochemical expression of p53 [13]. Another analysis of ASCP DNA reported that the squamous component was aneuploid and tetraploid, and that the adenocarcinoma component was diploid; the non-diploid pattern predicted an aggressive course [14]. More recently, a study of gene fusion in rare aggressive PC variants (*n* = 19), including ASCP (*n* = 7) [26], found that the frequency of *ERG* fusion in rare PC variants was similar to that of conventional PC (47%); that ERG immunohistochemistry for detecting rearrangement is less sensitive for variant histology than conventional PC; and that adenosquamous and sarcomatoid variants can particularly occur together [26].

Liquid biopsy using, for example, CTCs and cell-free DNA is an emerging topic in oncology [27–29]. Notably, Antonarakis et al. reported that detection of androgen-receptor splice variant 7 (AR-V7) in CTCs from patients with castration-resistant PC might be associated with resistance to enzalutamide and abiraterone [28]. More recently, Okegawa et al. analyzed CTCs from 98 patients with castration-resistant PC who received abiraterone or enzalutamide, and reported that both the presence of CTC clusters and AR-V7^+^ CTC clusters were important predictors of drug response and disease outcome [29]. However, no study has performed CTC analysis of ASCP because of its extreme rarity. The CTC analysis of the present case showed 5 CK^+^ CTCs, 14 CK^−^ CTCs, and 8 CTC clusters per 10 mL (Table 1A). An early study that assessed CTCs in metastatic castration-resistant PC showed that patients with ≥ 5 CTCs/7.5 mL (6.7 CTCs/10 mL) had significantly worse overall survival [27]. In addition, the aforementioned study by Okegawa et al. reported that the presence of CTC clusters is a predictor of worse outcome. Furthermore, as many as 14 CK^−^ CTCs per 10 mL were detected in the present case: CK^−^ CTCs are believed to be derived from epithelial–mesenchymal transition and to be thus more invasive and mobile than CK^+^ CTCs; our novel CTC detection device enabled the detection of CK^−^ CTCs, which was difficult by conventional devices like CellSearch®. Collectively, the above CTC results might reflect the patient’s advanced disease stage and aggressive nature of ASCP.

On the genomic analysis of CTCs in the present case, the NGS identified 14 mutations in 8 oncogenes or tumor suppressor genes: *PIK3CB*, *APC*, *CDKN2A*, *PTEN*, *BRCA2*, *RB1*, *TP53*, and *CDK12*. Of these14 variants, 9 (64%) were detected on CK^−^ CTCs and 5 (36%) were detected on CK^+^ CTCs. Furthermore, 5 of 14 (36%) mutations showed a high (> 20%) allele frequency (Table 1B). As suggested in a previous report [13], *TP53* mutations were identified in 5 CTCs along with other common oncogene or tumor suppressor gene mutations. Notably, 4 of 5 *TP53* mutations were classified as “likely pathogenic (LP)” variants. Meanwhile, the other mutation of *TP53* with an allele frequency of 28.9% (> 20%) was an intronic variant which was classified as a variant of uncertain significance (VUS). However, several studies have reported that intronic variants of the *TP53* gene can be associated with increased risk of developing cancer or worse survival outcomes by regulating gene expression [31, 32]. Similarly, intronic variants with a high (> 20%) allele frequency were identified in the *PIK3CB* and *CDKN2A* genes. Aside from these, it should be noted that a stop-gain variant classified as “LP” with a remarkably high (52.6%) allele frequency was detected in the *RB1* gene on a CK^−^ CTC. On the other hand, given the *BRCA2* mutation with a high (> 20%) allele frequency (albeit a VUS), Olaparib might have been effective for the present case, although the patient passed before it could be used. Because ASCP currently lacks effective treatment, its prognosis is dismal, with a median cancer-specific survival period of 16 months [1, 2]. Although a recent case report suggested the feasibility and possible efficacy of nivolumab (with or without radiotherapy) for ASCP [24], further evidence is warranted. Therefore, use of molecular-targeted agents based on liquid biopsy findings might be a promising treatment strategy for ASCP.

This study has several limitations. Since the detection capability of liquid biopsy is currently developing [30], its genomic analysis should be less comprehensive than the conventional tissue biopsy. We therefore decided to present all 14 mutations, including intronic variants, detected by the Ion Ampliseq™ Cancer Hotspot Panel v2 (Thermo Fisher Scientific). The panel can survey hotspot regions of 50 oncogenes and tumor suppressor genes, which, however, might be insufficient as a comprehensive genomic profiling. Furthermore, variants with a low allele frequency could include both false-positive results (sequencing artifacts) and true variants which were detected in a small fraction of the tumoral tissue. In addition, although a previous study suggested the clinical relevance of DNA ploidy in ASCP [14], this study did not assess the DNA ploidy.

In summary, we present a case of ASCP developed after primary ADT for Gleason grade group 5 PC and report results of CTC analysis and genomic analysis of CTCs in an ASCP patient for the first time. Although the prognosis of ASCP is currently dismal because of a lack of effective treatment, genomic analysis of CTCs might offer a potentially effective treatment, leading to improved survival.

## Data Availability

The datasets used and/or analyzed during the current study are available from the corresponding author (ST) on reasonable request.

## Abbreviations

ADT: Androgen deprivation therapy
AR-V7: Androgen-receptor splice variant 7
ASCP: Adenosquamous carcinoma of the prostate
CK: Cytokeratin
CT: Computed tomography
CTC: Circulating tumor cell
DAPI: Diamidino-2-phenylindole
LP: Likely pathogenic
NGS: Next-generation sequencing
PC: Prostate cancer
PSA: Prostate-specific antigen
SEER: Surveillance epidemiology and end results
VUS: Variant of uncertain significance
WGA: Whole-genome amplification

## References

[1] Bronkema C, Arora S, Sood A (2020). Rare histological variants of prostate adenocarcinoma: a national cancer database analysis. J Urol.

[2] Marcus DM, Goodman M, Jani AB, Osunkoya AO, Rossi PJ (2012). A comprehensive review of incidence and survival in patients with rare histological variants of prostate cancer in the United States from 1973 to 2008. Prostate Cancer Prostatic Dis.

[3] Thompson GJ (1942). Transurethral resection of malignant lesions of the prostate gland. JAMA.

[4] Parwani AV, Kronz JD, Genega EM, Gaudin P, Chang S, Epstein JI (2004). Prostate carcinoma with squamous differentiation: an analysis of 33 cases. Am J Surg Pathol.

[5] Wang J, Wang FW, Lagrange CA, Hemstreet GP (2010). Clinical features and outcomes of 25 patients with primary adenosquamous cell carcinoma of the prostate. Rare Tumors..

[6] Bennett RS, Edgerton EO (1973). Mixed prostatic carcinoma. J Urol.

[7] Accetta PA, Gardner WA (1983). Adenosquamous carcinoma of prostate. Urology.

[8] Saito R, Davis BK, Ollapally EP (1984). Adenosquamous carcinoma of the prostate. Hum Pathol.

[9] Moyana TN (1987). Adenosquamous carcinoma of the prostate. Am J Surg Pathol.

[10] Devaney DM, Dorman A, Leader M (1991). Adenosquamous carcinoma of the prostate: a case report. Hum Pathol.

[11] Ishigooka M, Yaguchi H, Tomaru M, Sasagawa I, Nakada T, Mitobe K (1994). Mixed prostatic carcinoma containing malignant squamous element. Reports of two cases. Scand J Urol Nephrol.

[12] Gattuso P, Carson HJ, Candel A, Castelli MJ (1995). Adenosquamous carcinoma of the prostate. Hum Pathol.

[13] Orhan D, Sak SD, Yaman O, Tulunay O, Gögŭş O (1996). Adenosquamous carcinoma of the prostate. Br J Urol.

[14] Bassler TJ, Orozco R, Bassler IC, Boyle LM, Bormes T (1999). Adenosquamous carcinoma of the prostate: case report with DNA analysis, immunohistochemistry, and literature review. Urology.

[15] Turhan OI, Aydin NE, Sariyüce O (1999). Adenosquamous carcinoma of the prostate. Int Urol Nephrol.

[16] Kim YW, Park YK, Park JH (1999). Adenosquamous carcinoma of the prostate. Yonsei Med J.

[17] Helal M, Diaz JI, Tannenbaum A, Greenberg H, Lockhart J (2000). Postradiation therapy adenosquamous cell carcinoma of the prostate. Prostate Cancer Prostatic Dis.

[18] Egilmez T, Bal N, Guvel S, Kilinc F, Ozkardes H (2005). Adenosquamous carcinoma of the prostate. Int J Urol.

[19] Baydar DE, Kosemehmetoglu K, Akdogan B, Ozen H (2006). Prostatic adenosquamous carcinoma metastasizing to testis. ScientificWorldJournal.

[20] Gao X, Wang HF, Li Y (2013). Treatment of bladder invasive adenosquamous carcinoma of the prostate: radical cystoprostatectomy. Chin Med J (Engl).

[21] Mishra S, Goel H, Awasthi N, Puri A, Mahapatra R, Pal DK (2014). Primary adenosquamous carcinoma of the prostate: a rare aggressive tumor. Clin Genitourin Cancer.

[22] Zhang Z, Wang Y, Zhao Q (2014). Mixed adenocarcinoma, sarcomatoid carcinoma and adenosquamous carcinoma of the prostate: a case report. Oncol Lett.

[23] Acosta AM, Kajdacsy-Balla A, Groth JV (2017). The squamous cells of adenosquamous carcinoma (ASCC) of the prostate might represent a terminally differentiated quiescent component: immunohistochemical evidence from a case of ASCC with pleomorphic giant tumor cells. Appl Immunohistochem Mol Morphol.

[24] Eze C, Manapov F, Gratzke C (2018). Concurrent radiotherapy and nivolumab in metachronous metastatic primary adenosquamous-cell carcinoma of the prostate. Eur J Cancer.

[25] Hennessey A, Buller D, Sama S, Girard E, Clement J, Ristau BT (2019). Case report: adenosquamous carcinoma of the prostate with greater than 20 month response to multimodal therapy. Urol Case Rep..

[26] Alhamar M, Tudor Vladislav I, Smith SC (2020). Gene fusion characterisation of rare aggressive prostate cancer variants-adenosquamous carcinoma, pleomorphic giant-cell carcinoma, and sarcomatoid carcinoma: an analysis of 19 cases. Histopathology.

[27] de Bono JS, Scher HI, Montgomery RB (2008). Circulating tumor cells predict survival benefit from treatment in metastatic castration-resistant prostate cancer. Clin Cancer Res.

[28] Antonarakis ES, Lu C, Wang H (2014). AR-V7 and resistance to enzalutamide and abiraterone in prostate cancer. N Engl J Med.

[29] Okegawa T, Ninomiya N, Masuda K, Nakamura Y, Tambo M, Nutahara K (2018). AR-V7 in circulating tumor cells cluster as a predictive biomarker of abiraterone acetate and enzalutamide treatment in castration-resistant prostate cancer patients. Prostate.

[30] Morimoto A, Mogami T, Watanabe M (2015). High-density dielectrophoretic microwell array for detection, capture, and single-cell analysis of rare tumor cells in peripheral blood. PLoS ONE.

[31] Wang-Gohrke S, Weikel W, Risch H (1999). Intron variants of the p53 gene are associated with increased risk for ovarian cancer but not in carriers of BRCA1 or BRCA2 germline mutations. Br J Cancer.

[32] Smeby J, Sveen A, Eilertsen IA (2019). Transcriptional and functional consequences of TP53 splice mutations in colorectal cancer. Oncogenesis.

